# Olive Pomace Fly Ash as an Alternative Alkaline Activator for Electric Arc Furnace Slag for Sustainable Cementitious Materials

**DOI:** 10.3390/ma18030601

**Published:** 2025-01-28

**Authors:** Ana Muñoz-Castillo, Francisca Andrés-Castro, Miguel Ángel Gómez-Casero, Dolores Eliche-Quesada

**Affiliations:** 1 Department of Chemical, Environmental, and Materials Engineering, Higher Polytechnic School of Jaén, Campus Las Lagunillas, University of Jaén, s/n, 23071 Jaen, Spain; amcastil@ujaen.es (A.M.-C.); fac00019@red.ujaen.es (F.A.-C.); magomez@ujaen.es (M.Á.G.-C.); 2 Center for Advanced Studies in Earth Sciences, Energy and Environment (CEACTEMA), Campus Las Lagunillas, University of Jaén, s/n, 23071 Jaen, Spain

**Keywords:** alkaline-activated cements, electric arc steel slag, olive pomace fly ash, alternative alkaline activator, mechanical properties

## Abstract

This study analyzes the potential of olive pomace fly ash (OPFA) as an alternative alkaline activator for electric arc furnace slag (EAFS) in the manufacture of sustainable cementitious materials. Cements were prepared by replacing 30–50 wt% of EAFS with OPFA and compared with control cements activated with potassium hydroxide (KOH) at concentrations of 4 and 8 M. Cements were characterized by bulk density, water absorption, total porosity, compressive and flexural strength, as well as analytical techniques such as XRD, FTIR and SEM-EDS. The results reveal that the incorporation of 40 wt% OPFA provides optimum properties, reaching maximum compressive and flexural strengths of 20.0 MPa and 5.7 MPa, respectively, after 28 days of curing. These improvements are attributed to the increased formation of C,K-A-S-H gel, which incorporates Fe, the main reaction product that densifies the matrix and reduces porosity. However, 30 wt% OPFA provides insufficient alkali content, which limits the reaction, while excess alkali at 50 wt% OPFA reduces mechanical performance due to unreacted residues and increased interconnected porosity. Compared to KOH-activated cements, which achieve maximum flexural and compressive strengths of 4.4 and 9.5 MPa (EAFS/KOH-8M binders), the results confirm the potential of OPFA as an alternative activator, with significant sustainability advantages.

## 1. Introduction

The search for more sustainable building materials has led to research into alternative cementitious materials, such as alkaline-activated cements (AAC) or geopolymers, binders that could be more eco-efficient than Portland cement by minimizing environmental impacts such as CO_2_ emissions and energy consumption [1,2]. These materials are obtained by chemical activation of materials rich in silica (SiO_2_) and alumina (Al_2_O_3_) with high or low calcium (CaO) content with alkaline solutions. The main reaction product in the former is hydrated calcium aluminosilicate gel (C-A-S-H), while in the latter it is geopolymer gel or N-A-S-H gel [3]. 

The behavior of AAC depends on the precursor and activator used. The precursor can be from natural sources such as kaolin, clays or zeolite [4,5,6] or from artificial sources such as industrial wastes and by-products like blast furnace slag [7], fly ash [8], biomass ash [9], sewage treatment sludge [10], ceramic wastes [11] and ferrous and non-ferrous electric arc furnace slags [12], among other waste. The use of industrial waste and by-products versus natural sources as a source of aluminosilicates not only improves sustainability and reduces costs, but also drives technological innovation and strengthens the circular economy.

As for alkaline activators, they are substances that supply alkaline cations, which increase the pH level and facilitate the dissolution process [13]. The most commonly used are hydroxides (NaOH and KOH) and alkaline silicates (Na_2_SiO_3_ and K_2_SiO_3_) [14,15]. Hydroxides and liquid silicates present significant challenges for their application on an industrial scale due to their corrosive nature, high viscosity and the heat generated during handling [16]. Moreover, they constitute the most expensive component in the production of AAC [17]. These activators, in particular, sodium silicate, are mainly responsible for the environmental impact associated with AAC [17]. The production of alkaline activators is estimated to contribute approximately 60% of the greenhouse gas emissions associated with the manufacture of these cements, which is one of the main barriers to their widespread adoption [1].

A promising strategy to reduce the environmental impact of CSA is the production of alternative alkaline activators from industrial wastes and by-products. This enables the reuse and conversion of waste into high-value products, thus contributing to more sustainable and environmentally friendly construction [18]. 

There are several possibilities to find alternative activators to the commonly commercial ones, using agricultural and industrial wastes. These include alkali-based alternative activators, such as biomass ash.

Biomass combustion ash is presented as a promising alternative, as it may contain high concentrations of soluble alkaline compounds that act as activators [19].

In particular, ash from the olive oil industry, such as olive pomace ash, is of interest because of its abundance in olive oil-producing regions such as Spain.

Olive pomace is obtained as a by-product during the olive oil extraction process in olive oil mills. Wet pomace (60 wt% moisture) is produced specifically in hydraulic presses or mechanical extraction systems, together with the olive oil. This wet pomace must undergo drying to obtain the ‘orujillo’ or olive pomace, which is composed mainly of olive pulp with residual oil, crushed olive stones and a small quantity of water and residual oil [20].

Total olive oil production in the Mediterranean countries during the 2022/2023 marketing year reached approximately 2,760,000 tonnes [21]. For each tonne of olives processed, between 800 and 950 kg of wet olive pomace is obtained [22]. This quantity is expected to increase due to the rapid growth in global demand for olive oil.

The most widespread use of olive pomace is in industrial biomass boilers as a fuel in energy production plants [23], producing olive pomace ash as a residue. One of the applications of olive pomace ash is its use as a fertilizer or soil amendment due to its potassium and calcium content [24] and as an adsorbent in the treatment of industrial waste water [25].

Due to the high accumulation of these by-products in landfills, several studies have been carried out to evaluate their potential reuse in building materials. Their use as a replacement for clay in ceramic bricks has been investigated [26,27], as a replacement for sand in Portland cement mortars [28], and as a replacement for cement in mortars after the application of different processing methods to improve the properties of the mortar [29].

Ash from olive oil residues has enormous potential as an alkaline activator in the manufacture of alkaline-activated cements. Pinheiro et al., 2018 [30] studied the feasibility of using olive pit ash (OBA) as an alternative activator for mortars based on blast furnace slags (BFS). The results showed that mortars activated with OBA have similar or higher strengths compared to mortars obtained by activation of BFS activated with sodium or potassium hydroxides. Alonso et al., 2019 [31] investigated the use of 30 wt% olive biomass ash, biomass fly ash (OBFA) and bottom ash (OBBA) as potential alkaline activators in the preparation of alkaline-activated cements or blast furnace glassy slag geopolymers (70 wt%). Cements activated with OBFA and OBBA show mechanical strengths of 33 and 18 MPa, respectively, after 28 days of curing, indicating the potential of these ashes as an activator. Gómez-Casero et al., 2021 [32] evaluated the use of 30 wt.% olive pomace fly ash (OPFA) as an alkaline source for the activation of calcined clays (CCs) from Bailén (Jaén, Spain). The cements obtained showed a compressive strength of 1.3 MPa, slightly lower than that achieved with alkaline hydroxide activating solutions (3.0–3.9 MPa). These results suggest that OPFA can be used as an alkaline source for the activation of CC, achieving geopolymers with adequate physical and thermal properties, although with limited mechanical properties. Zehao Lei and Sara Pavia, 2024 [33] activated ground granulated blast furnace (GGBS) (40 wt%) with olive pit ash (60 wt%), also using auxiliary activators such as sodium carbonate and hydrated lime. The control mortars achieved compressive strengths of 15.43 MPa, increasing the compressive strength with the use of up to 10% sodium carbonate and up to 5% lime. 

This work studies the potential of using olive pomace fly ash (OPFA) in the alkaline activation of electric arc furnace slag (EAFS)-based cements. It examines the influence of the EAFS/OPFA ratio on the technological properties of the binder and makes a comparison with control cements activated with a KOH solution at 4 M and 8 M concentrations. It proposes the manufacture of cements using waste as raw materials, a sustainable solution with a lower environmental impact and reduced energy consumption compared to traditional cements, promoting a circular economy.

## 2. Materials and Methods

### 2.1. Raw Materials

The raw materials used in this study included electric arc furnace slag (EAFS), olive pomace fly ash (OPFA) and potassium hydroxide (KOH). 

**Figure 1:**
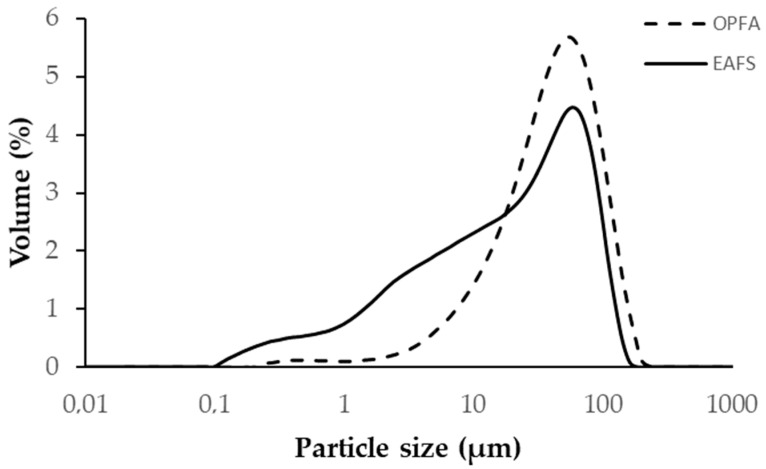
Particle size distribution of raw materials: electric arc furnace slag (EAFS) and olive pomace fly ash (OPFA).

**Figure 2:**
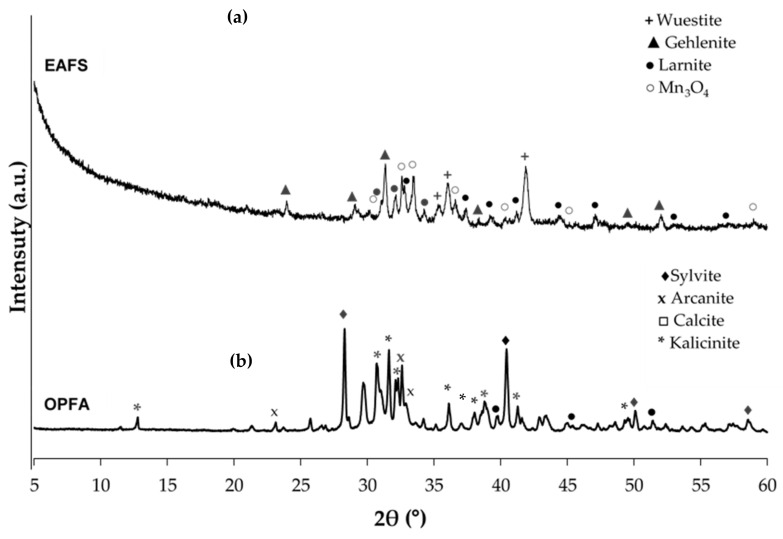
XRD of raw materials: (**a**) electric arc furnace slag (EAFS) and (**b**) olive pomace fly ash (OPFA).

**Figure 3:**
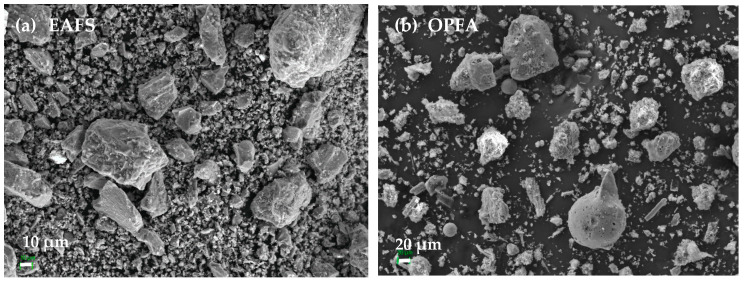
SEM micrographs of raw materials: (**a**) electric arc furnace slag (EAFS) and (**b**) olive pomace fly ash (OPFA).

EAFS was used as a precursor in all formulations and was supplied by Siderúrgica Sevillana (Alcalá de Guadaira, Seville, Spain). The material was supplied with a maximum particle size of 2 cm, so it was crushed in a jaw crusher and a ball mill, followed by a screening process to a size of less than 100 µm. The grain size distribution was determined by laser diffraction analysis with a Malvern Mastersizer 2000 diffractometer (Malvern, Worcestershire, UK), obtaining a value of d_90_ = 76.9 μm. The particle size distribution of the slag is presented in Figure 1. The chemical composition of the steel slags was determined using X-ray fluorescence (XRF), using Philips Magix Pro equipment (model PW-2440), Malvern, Worcestershire, UK) and the results are detailed in Table 1. The main compounds identified in the EAFS were CaO (30.89%), Fe_2_O_3_ (24.16%), SiO_2_ (17.29%) and Al_2_O_3_ (10.72%). The characterization of the crystalline phases present in the EAFS was performed by X-ray diffraction (XRD), using a PANalytical Empyrean system with PIXcel-3D detector (Malvern, Worcestershire, UK) and irradiation with Cu K X-rays (λ = 1.5406 Å), operating at 40 kV and 40 mA, with a 2θ range from 10° to 60° and a step of 0.02°. The identification of the crystalline phases was performed with HighScore 5.2 software. The results (Figure 2a) showed that the slags have a predominantly amorphous structure, with crystalline phases of wuestite (FeO), larnite (Ca_2_SiO_4_), gehlenite (Ca_2_Al(SiAl)O_7_) and small amounts of manganese oxide (Mn_3_O_4_). The morphological and particle size analysis of the EAFS particles was performed by scanning electron microscopy (SEM) using a Merlin Carl Zeiss instrument (Malvern, UK) complemented by energy-dispersive X-ray spectroscopy (EDS) using an Oxford Inca Energy 350X-MAX 50 (High Wycombe, UK). The accelerating voltage was set to 15 kV, with a beam current of 750 pA. The samples were mounted on aluminum supports and carbon coated using a JEOL JFC 1100 sputter coater (Akishima, Tokyo, Japan). The images obtained, shown in Figure 3a, revealed that the EAFS particles are irregular in shape and have a wide size distribution.

The OPFA used in this study was provided by the biomass plant Energía La Loma S.A. (Villanueva del Arzobispo, Jaén, Spain). This waste is generated by the combustion of olive pomace for the production of electricity. The ash used is fly ash, or particles carried by the exhaust gases out of the combustion chamber. The sample was dried at 105 °C for 24 h and then sieved to a particle size of less than 63 μm in order to increase its dissolution rate in water. The results of the particle size analysis indicate that the ash has a particle size d_90_ of 107.7 μm, and its particle size distribution is presented in Figure 1. Regarding its chemical composition (Table 1), high contents of K_2_O (52.08%), followed by SO_3_ (6.81%) and CaO (5.33%), are observed. X-ray diffraction (XRD) analysis identified the main crystalline phases present in the sample, which include sylvite (KCl), arcanite (K_2_SO_4_), calcite (CaCO_3_) and potassium bicarbonate, and kalicinite (KHCO_3_), as shown in Figure 2b. Morphological analysis of the OPFA particles, shown in Figure 3b, revealed a variety of shapes and sizes, highlighting irregular particles, some spherical and others with porous structure.

### 2.2. Cement Preparation

Alkaline-activated cements were manufactured by substituting the EAFS precursor with different amounts of OPFA (30–50 wt%) used as an alkaline activator. Both solids were mixed in a Proeti mixer for 2 min until homogenization. Water was then added and mixed for 2.5 min. The pastes were manufactured with a water/solid ratio, w/s = 0.26. The mixture was deposited in 60 × 10 × 10 mm rectangular stainless steel molds, beaten 60 times on a shaking table to remove bubbles for better compaction, screeded, sealed and cured at 80 °C for 7 days, after which it was demolded. The cements were named EAFS/xOPFA, where ‘x’ is the OPFA substitution content (x = 30, 40 and 50 wt%).

Control cements were obtained by alkaline activation of EAFS with potassium hydroxide solution at 4 M and 8 M concentrations. These pastes were named EAFS/KOH-yM, where ‘y’ is the molarity value of the alkaline solution (y = 4 and 8 M). Twelve samples were manufactured from each composition.

The composition of the manufactured pastes can be seen in Table 2.

### 2.3. Alkaline-Activated Cements Characterization

Bulk density and water absorption were evaluated following Archimedes’ principle, according to the procedure established in the UNE-EN 1015-10 standard [34]. On the other hand, the real density of the cements was determined using the pycnometer method, using ethanol as a solvent. From the values obtained for the real density and the bulk density, the total porosity was calculated by applying Equation (1).

(1) $$Total\;porosity\left(\%\right)=\left(1-\frac{Bulk\;density}{true\;density}\;\right)\times 1000$$

Flexural and compressive strength were evaluated according to UNE-EN 1015-11:2000/A1:2007 [35], using the MTS Insight 5 testing machine (Eden Prairie, MN, USA) and the MTS 810 Material Testing Systems laboratory press (Eden Prairie, MN, USA), respectively. The displacement speed used was 1.0 mm/min for the bending tests and 2.0 mm/min for the compression tests. Six specimens were used to determine the physical and mechanical properties at each curing age. The degree of reaction was determined to quantify the fraction of material that was not solubilized after exposure to an HCl solution (1:20). One gram of cement sieved to a particle size < 100 μm was placed in an HCl solution (1:20) and stirred for 3 h. The solution was then filtered through a filter paper with a pore size between 15 and 20 μm. The insoluble residue was washed with deionized water to neutral pH, dried, weighed (*P_i_*) and calcined at 1000 °C for one hour and reweighed (*P_f_*). 

This parameter reflects the amount of unreacted binder during the alkaline activation process. The products resulting from the reaction, corresponding to the formation of the geopolymer, were solubilized in the HCl solution, while the insoluble fraction represents the unreacted material, which is not part of the geopolymer matrix [36]. 

The degree of reaction is calculated by Equation (2):

(2) $$Reaction\;degree=100-\left(\frac{Pi}{Pf}\right)*100$$

Fourier transform infrared spectroscopy (FTIR) spectra of the raw materials and cements were recorded using a Bruker Tensor 27 FTIR spectrometer (Billerica, MA, USA) using the attenuated total reflection (ATR) method. Spectra were collected in the range 4000–400 cm^−1^, with a resolution of 4 cm^−1^ in the absorbance mode. The mineralogical composition of the cements was determined by XRD, while the microstructure was analyzed by SEM-EDS. The equipment and operating conditions used were the same as for the raw materials.

## 3. Results and Discussion

### 3.1. Bulk Density, Water Absorption and Total Porosity

The bulk density of the EASF/xOPFA cements is similar, obtaining values between 1830 kg/cm^3^ for EAFS/30OPFA cements and 1879 kg/cm^3^ for EAFS/40OPFA cements after 28 days of curing (Table 3). The control cements present higher bulk densities, with values of 1940 kg/m^3^ and 2039 kg/m^3^ for EAFS/NaOH-4M and EAFS/NaOH-8M, respectively, after 28 days of curing. The higher bulk density of the control cements could be related to the higher real density of the EAFS, 3245 kg/m^3^. The real density of the alternative activator, OPFA, is much lower than that of the EAFS precursor, 2240 kg/m^3^, which justifies the lower bulk density of the binders. However, there is no decrease in bulk density with increasing incorporation of OPFA, obtaining slightly higher values with the incorporation of 40 wt% ash. This could be related to the higher formation of more compact cementitious products in the EASF/40OPFA cements, according to the reaction degree data (Table 3).

The water absorption and total porosity of the binders follow a trend opposite to the bulk density. The water absorption and total porosity values for EAFS/30OPFA cements were 13.0 and 34.3%, respectively. With the use of 40 wt% OPFA, EAFS/40OPFA cements resulted in lower values of 8.2 and 24.5%, respectively. An increase in OPFA incorporation resulted in a slight increase in both properties, increasing to 9.4 and 27.6%, respectively. A higher water absorption and total porosity are observed when a lower amount of OPFA is used, which could be due to the filling effect of the OPFA. The undissolved ash could act as a filler, decreasing the porosity.

The EAFS/NaOH-yM control cements, although presenting higher bulk densities compared to the cements using OPFA as an alternative activator, show similar values of water absorption and total porosity: 14.34% and 25.0% for EAFS/NaOH-4M and 11.51% and 23.5% for EAFS/NaOH-8M, respectively. The increase in these properties could be related to the higher fluidity of the samples using the commercial activator, since the water/solid ratio is kept constant in this study. This suggests that a proportion of the water incorporated in the EAFS/NaOH-yM cements is not consumed during the hydration reactions, resulting in a higher porosity of the cementitious matrix.

For all cements, there was a slight increase in bulk density and a decrease in water absorption and total porosity with curing time. This behavior can be attributed to a slight advance of alkaline activation reactions leading to the formation of K-A-S-H and C-A-S-H gel products, which fill the pores of the matrix.

### 3.2. Bending and Compressive Strength

**Figure 4:**
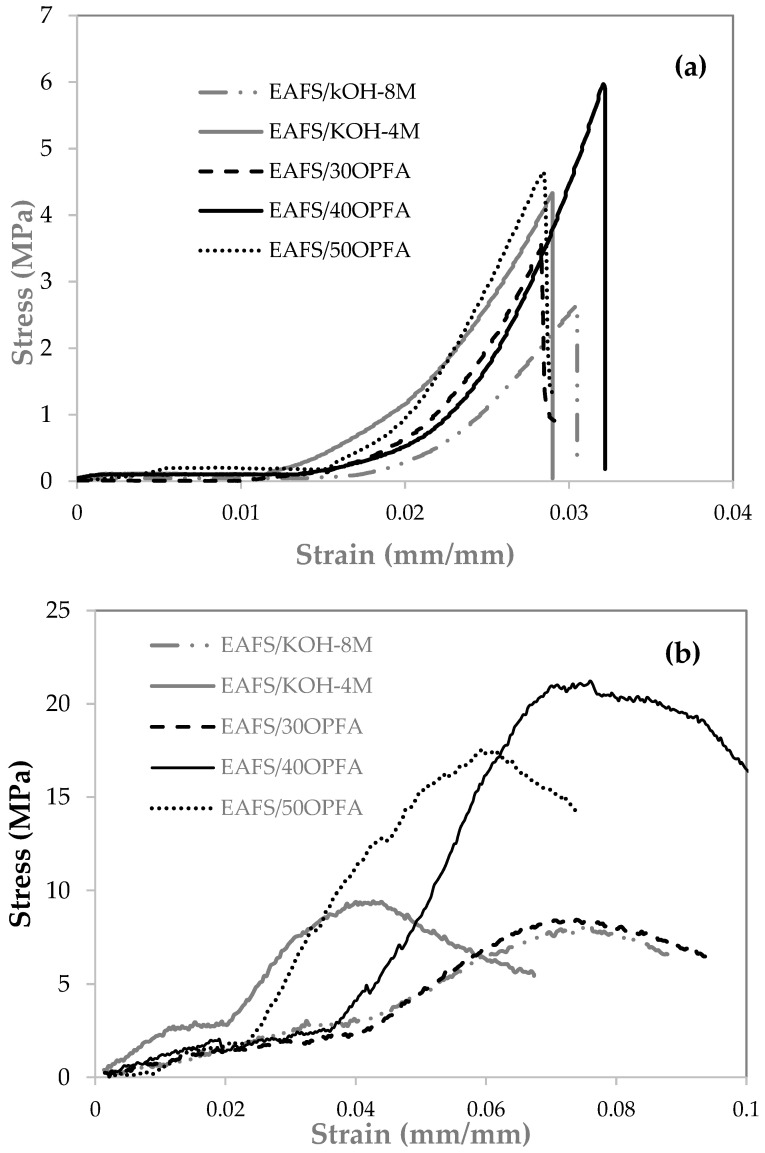
Stress–strain curves of (**a**) flexural strength and (**b**) compressive strength of the cements activated with KOH and OPFA at 28 days of curing.

Figure 4 shows flexural and compression curves for one of the specimens of each alkaline-activated cement manufactured. It can be seen that the load–deformation curves of the flexural tests show typical brittle behavior in alkali-activated cements, with an initial linear increase up to the maximum load, followed by an abrupt drop. The compression curves reveal a higher ductility compared to the flexural tests, with a more evenly distributed load capacity before fracture. Cements activated with OPFA show a high initial slope, indicating higher stiffness in the elastic phase.

**Figure 5:**
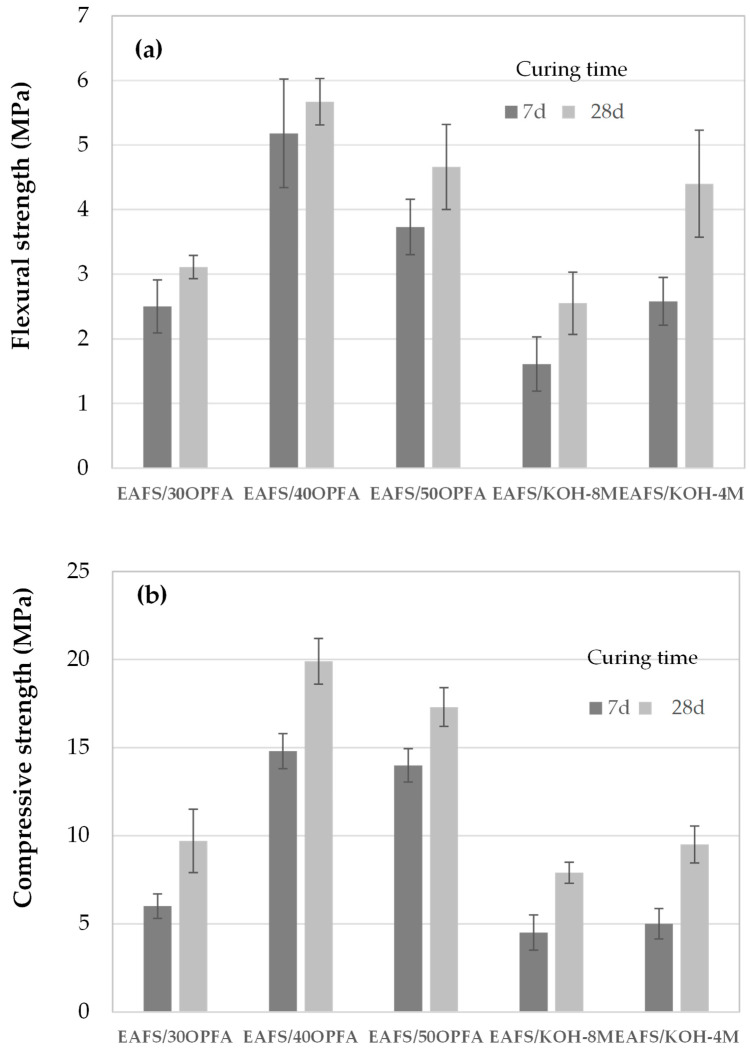
(**a**) Flexural and (**b**) compressive strength of EAFS-based binders activated with OPFA and KOH as a function of curing time.

The mechanical strength in terms of flexural and compressive strengths after 7 and 28 days of curing of the alkaline-activated cements are shown in Figure 5. The flexural and compressive strengths after 28 days of curing of EAFS/xOPFA cements were 3.1, 5.7 and 4.7 MPa and 9.7, 19.9 and 17.3 MPa, respectively for EAFS/30OPFA, EAFS/40OPFA and EAFS/50OPFA. Therefore, the flexural and compressive strength increases with the addition of OPFA, up to 40 wt%, and then decreases. OPFA contains a high concentration of water-soluble compounds, such as potassium (K_2_O) and sulphates (SO_3_), which contribute to the increase in pH and favor the dissolution of Ca^2+^, Fe^3+^, Si^4+^ and Al^3+^ ions present in the EAFS precursor. The reaction between the released ions generates cementitious gels such as C,K,Fe-A-S-H, which are responsible for the cohesion and the development of mechanical properties. The increase in mechanical strength could be due to a higher degree of reaction, resulting in a higher amount of reaction products, C,K,Fe-A-S-H gel, which results in a filling of voids and pores during the alkaline activation reaction, leading to a slightly denser structure that contributes to the development of higher strength. With 40 wt% OPFA and 60 wt% EAFS, it could be had the right amount of alkali present in OPFA, which favors maximum dissolution of the EAFS precursor resulting, as the reaction degree results indicate (Table 3), in a higher gel structure, leading to high strength in the binders.

The incorporation of amounts higher than 40 wt% of OPFA could lead to an excess of alkali not intervening in the activation of the EFAS precursor, hindering the alkaline activation reaction and causing a decrease in mechanical strength [37]. Thus, a 30 wt% incorporation of OPFA results in the presence of unreacted EAFS due to an insufficient amount of alkali, while a 50 wt% amount of OPFA results in a slight excess of alkali and the presence of unreacted OPFA. In both cases, the matrix shows a higher amount of voids and pores, as well as a lower degree of reaction, resulting in cements with lower mechanical properties.

The flexural and compressive strength values when the EAFS are activated with KOH solutions were, after 28 days of curing, 2.6 and 7.9 MPa for EAFS/NaOH-4M binders and 4.40 and 9.5 MPa for EAFS/NaOH-8M cements, respectively, presenting similar values to those obtained with the activation of 30 wt% OPFA, EAFS/30OPFA binders. The slight increase in the mechanical properties of EAFS/NaOH-8M cements could be related to the higher bulk density and lower water absorption and total porosity. A 4 M NaOH concentration leads to a slower dissolution rate of the precursor, EAFS, resulting in a lower matrix density due to insufficient dissolution of Si^4+^, Al^3+^, Ca^2+^ and Fe^3+^ ions, essential for gel formation, resulting in a more porous matrix, which contributes to a lower mechanical strength in terms of flexural and compressive strength.

It is observed that the compressive strength increases as the SiO_2_/Al_2_O_2_O_3_ molar ratio increases from 2.83 to 2.88 (Table 1) in EAFS/30OPFA and EAFS/40OPFA cements, respectively, which is probably due to the formation of denser, shorter and stronger Si-O-Si bonds compared to Si-O-Al or Al-O-Al bonds [38].

However, a further increase in the SiO_2_/Al_2_O_3_ ratio up to 2.94 in EAFS/50OPFA cements causes a decrease in strength, attributed to an increase in the alkali activator/slagylates ratio, resulting in a more porous microstructure. This porosity, along with the appearance of microcracks and microstructural inhomogeneities [39], decreases reactivity and gel-forming ability. In addition, an excess of Si/Al reduces the dissolution rate of aluminosilicate precursors and hinders structural reorganization and gel densification. On the other hand, the low SiO_2_/Al_2_O_3_ ratios of 4M and 8M KOH-activated cements, 2.74, result in a high amount of interconnected pores and voids, generated by unreacted slags, which significantly compromises the compressive strength [40].

The flexural and compressive strength values obtained are slightly lower than those obtained by other authors using biomass ash to activate blast furnace slag, which is more reactive than electric arc furnace slag.

Pinheiro et al., 2018 [30] activated blast furnace slag (BFS) with olive stone biomass ash (OBA) and obtained flexural and compressive strength values of 6.5 and 26.0 MPa with 20% replacement of BFS by OBA, and optimum values of 6.0 and 31.0 MPa with 30% replacement. For KOH-activated mortars, the highest flexural and compressive strengths of 3.7 and 28.6 MPa are obtained for BFS mortars activated with 8 M KOH solution. Alonso et al., 2019 [31] studied the activation of glassy blast furnace slags with 30 wt.% olive biomass fly ash (OBFA) and olive biomass bottom ash (OBBA). The pastes obtained reached compressive strengths of 33 and 18 MPa, respectively, after 28 days of curing. Soriano et al., 2020 [41] investigated the use of almond shell biomass (ABA) ash (20 wt% substitution or addition) as an alkali source in blast furnace slag mortars. The addition mortar exhibited compressive strengths of 36.4 MPa after 7 days of curing versus 34.7 MPa for the substitution mortar. Payá et al. [42] studied the activation of blast furnace slag (BFS) with olive stone biomass ash (OBA) to prepare compacted dolomitic soil blocks. The stabilization of the dolomitic soil was carried out by adding 10% BFS, 4% OBA and 8% water, and after 120 days of curing, the blocks obtained a compressive strength of 27.8 MPa.

### 3.3. Characterization of Hydration Products

**Figure 6:**
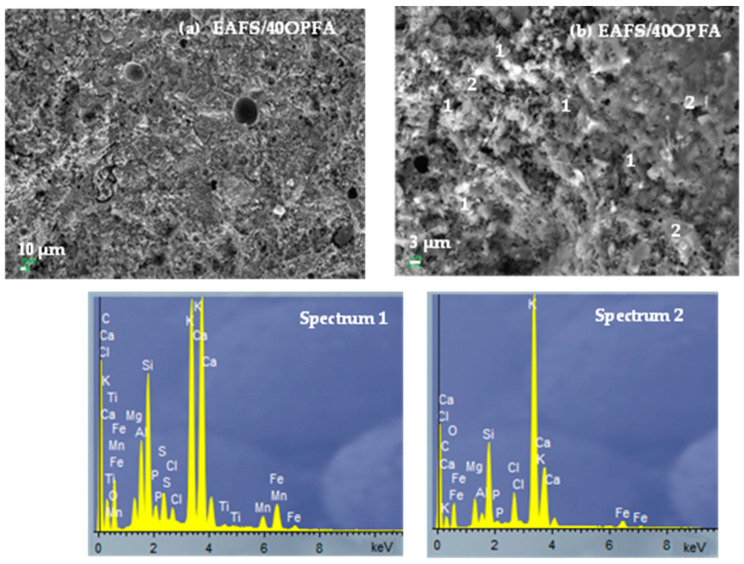
SEM-EDS analysis of EAFS/40OPFA cements: (**a**) 500× and (**b**) 3000×.

**Figure 7:**
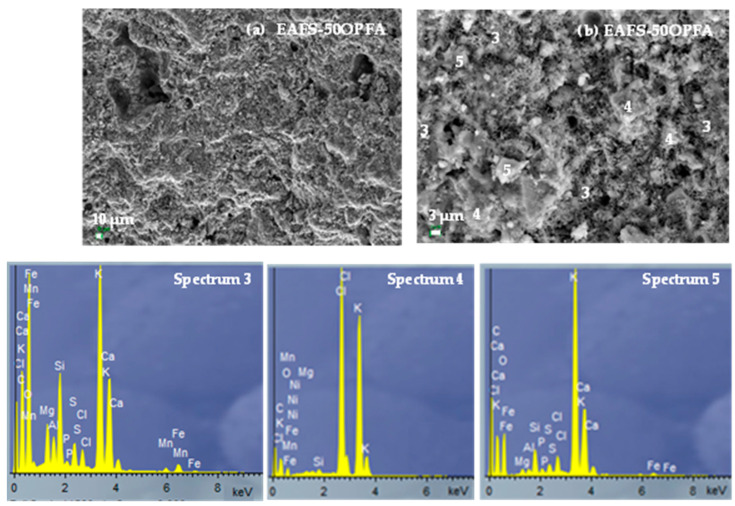
SEM-EDS analysis of EAFS/50OPFA cements: (**a**) 500× and (**b**) 3000×.

The microstructure of the hydrated products after 28 days of curing of the alkaline-activated cements (EAFS-40OPFA and EAFS-50OPFA) was analyzed by SEM-EDS (Figure 6 and Figure 7). A similar densification of the binders can be observed, although slightly higher in the EAFS-40OPFA sample. In both cements, the presence of pores is observed, although of smaller size in the cements incorporating 40 wt% OPFA. In the EAFS-40OPFA cements, the formation of gel rich in calcium (22.7%), potassium (18.9%) and iron (6.4%) is observed, as in the EAFS-50OPFA cements that present amounts of calcium (25.1%), potassium (11.8%) and iron (3.4%) (Spectrum 3), indicating a lower incorporation of potassium in the gel (C,K,Fe-A-S-H). This could indicate an excess of OPFA in the system, which prevents the K_2_O present from completely dissolving during the reaction process. This is corroborated by the presence of unreacted ash particles in both cements (Spectrum 2 and Spectrum 4), more abundant in the EAFAS-50OPFA binders, as well as the presence in the latter of sylvite (KCl) crystals, the crystalline phase of OPFA (Spectrum 5). The presence of unreacted particles and unreacted residual phases leads to a higher interconnected porosity and a reduction of the observed compressive strength.

**Figure 8:**
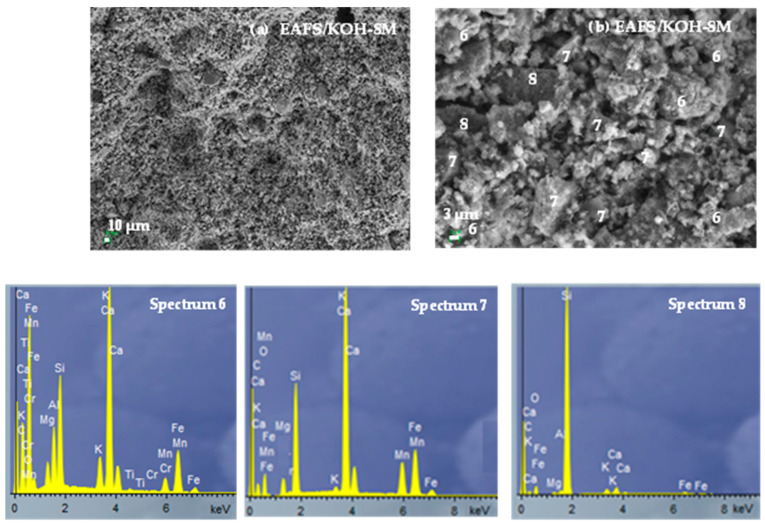
SEM-EDS analysis of EAFS/KOH-8M cements: (**a**) 500× and (**b**) 3000×.

The microstructure of the EAFS activated with KOH 8 M solution is presented in Figure 8. A dense matrix with the presence of both closed and open porosity can be observed. EDS analysis indicates the presence of gel rich in calcium (21.8%), potassium (10.2%) and iron (9.3%) (C,K,Fe-A-S-H) (Spectrum 6). Angular particles of unreacted slag (Spectrum 7) and particles of unreacted quartz present in the EAFS residue (Spectrum 8) are also observed. The amount of silica in the gel of EAFS-activated cements is 9.3 and 8.0 for EAFS-40OPFA and EAFS-50OPFA cements, respectively, and 7.8% for 8M KOH-activated cements, indicating the same nature of the gel formed, regardless of the activator used.

**Figure 9:**
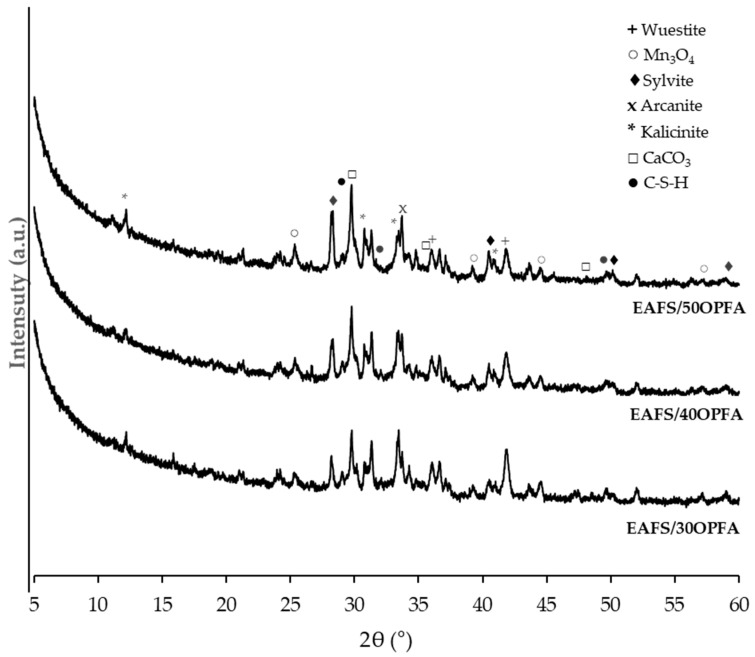
XDR patterns of EAFS-alkali-activated cements activated with OPFA at 28 days of curing time.

Figure 9 shows the results of the XRD analysis of the OPFA-activated cements after 28 days of curing. All the OPFA-activated pastes show similar diffraction patterns, with only differences in the intensity of the diffraction peaks. Diffraction peaks corresponding to the EAFS and OPFA raw materials can be observed, although with lower intensity than in the precursors, indicating partial solubility. Therefore, the EAFS and OPFA particles have partially dissolved and reacted, and the cements obtained show a certain degree of crystallinity. Thus, peaks corresponding to the wuestite and Mn_3_O_4_ crystalline phases present in the EAFS and the acarnite (K_2_SO_4_) and sylvite (KCl) crystalline phases present in the OPFA activator are observed. In addition, peaks corresponding to the carbonation of the cements, calcium carbonate and potassium carbonate, due to the reaction of Ca and K with atmospheric CO_2,_ potassium and calcium carbonate are also present in the OPFA activator. Small peaks due to the formation of the C-S-H gel, due to its amorphous character, are observed. In all specimens, a baseline deviation between 25° and 40° 2θ is observed, indicating that during the alkaline activation reaction, the K-A-S-H geopolymer gel that provides strength to the binder has formed [43]. This amorphous halo is observed more clearly in cements activated with OPFA, EAFS/40OPFA and EAFS/50OPFA, indicating a greater alkaline activation in these binders, corroborating the compressive strength results.

**Figure 10:**
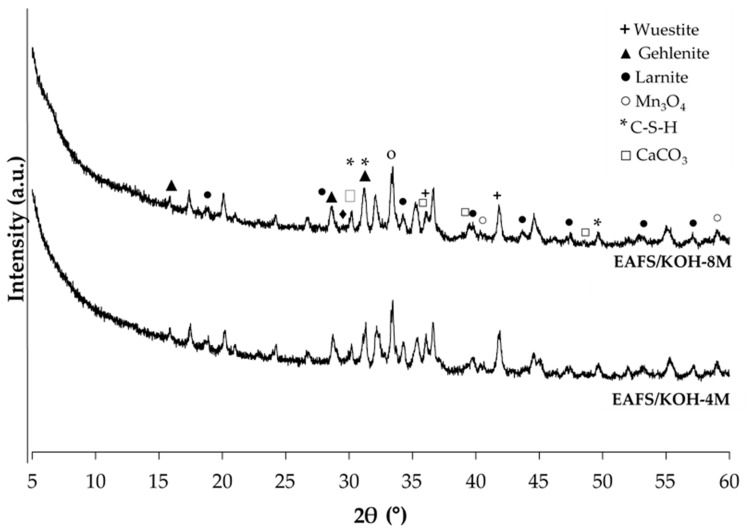
XDR patterns of EAFS-alkali-activated cements activated with KOH at 28 days of curing time.

The cements activated with KOH (Figure 10) also present similar XRD patterns, where diffraction peaks of the EASF precursors wuestite, Mn_3_O_4_, larnite and gehlenite are observed, as well as the appearance of calcium carbonate, due to the calcium present in the EAFS residue reacting with atmospheric CO_2._ The formation of C-S-H gel and K-A-S-H geopolymeric gel is also observed in smaller proportions, the latter due to a smaller halo between 25° and 40° 2θ, which could justify the lower mechanical strength of these binders. 

**Figure 11:**
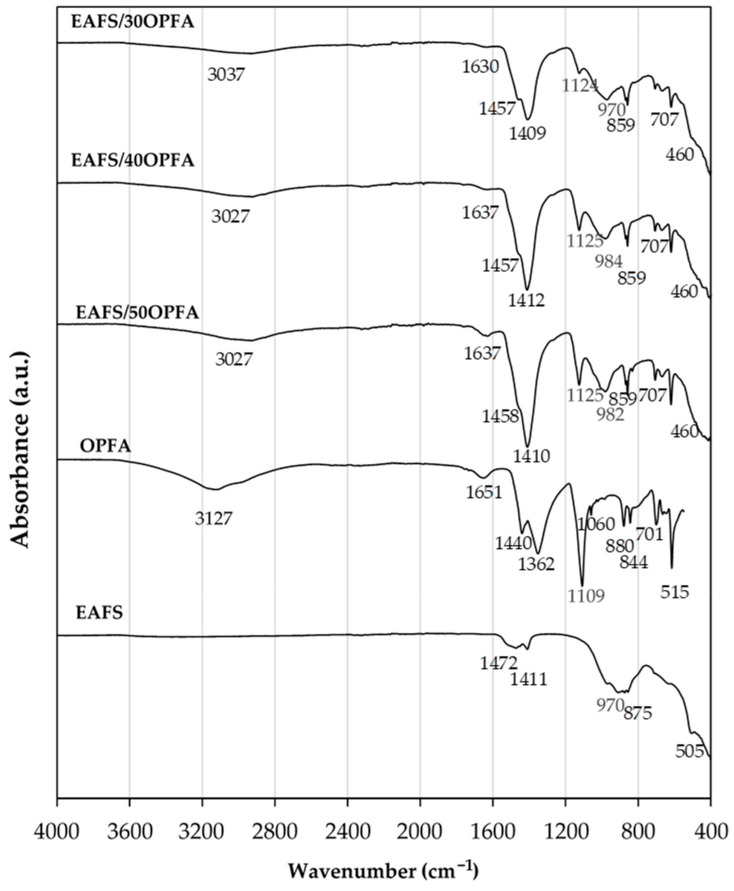
FTIR spectra of precursor (EAFS), alternative activator (OPFA) and the alkali-activated cements EAFS-xOPFA at 28 days of curing time.

**Figure 12:**
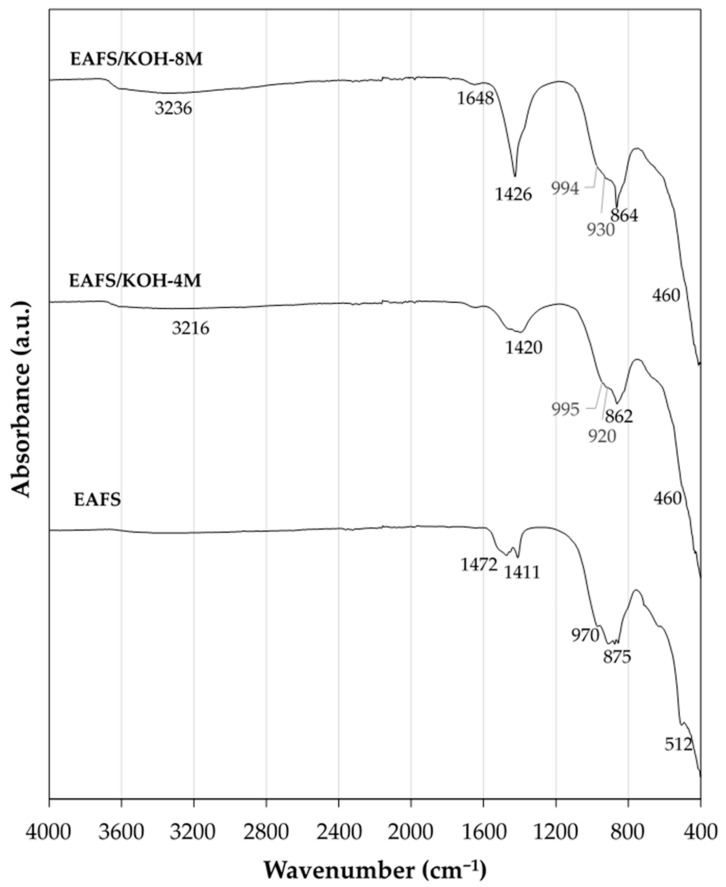
FTIR spectra of precursor (EAFS) and the alkali-activated cements activated with KOH at 28 days of curing time.

The FTIR spectra of the OPFA-activated cements are shown in Figure 11, incorporating the FTIR of the EAFS precursor and the OPFA activator for reference. In all the FTIR spectra of the cements, an intense peak centered at 1125 cm^−1^ is identified, accompanied by bands centered between 970 and 982 cm^−1^. On the other hand, the FTIR spectra of cements activated with KOH solutions at 4 M and 8 M concentrations (Figure 12) present a broad band centered at approximately 930 cm^−1^ and 920 cm^−1^, respectively, with a barely perceptible shoulder around 1000 cm^−1^. These bands are associated with the symmetric stretching vibration of the Si-O-T bond (T = Si or Al). The bands observed at lower wavenumbers indicate the predominant formation of calcium silicate hydrates (C-S-Hs) and calcium aluminosilicate hydrates (C-A-S-Hs) [44], with a low degree of geopolymerization, whereas the bands centered at higher wavenumbers are attributed to the presence of a K-A-S-H-type geopolymeric gel [45]. These results confirm the formation of a hybrid C,K-A-S-H gel, resulting from the coexistence of the K-(A)-S-H and C-(A)-S-H gels generated during the alkaline activation process [46,47]. Additionally, the presence of Fe in the EAFS suggests a partial oxidation to Fe³⁺ during activation, allowing its incorporation into the silicate network, replacing Si-O-Si bonds with Si-O-Fe [48]. This results in higher gel formation in OPFA-activated cements, especially in EAFS/40OPFA and EAFS/50OPFA composites, as confirmed by compressive strength data.

Additionally, a band at 707 cm^−1^, associated with asymmetric stretching of the Si-O-Si and Al-O bonds, is identified in the OPFA-activated cements. This observation confirms that the geopolymerization process has taken place [49].

On the other hand, the shoulder detected around 460 cm^−1^ in cements is attributed to the deformation vibration of Si-O-Si bonds, which is related to the presence of C-S-H gel [50]. 

The broad absorption bands located between 3500 and 2700 cm^−1^ in OPFA- and KOH-activated cements are associated with the stretching vibrations of the -OH bond (~3400 cm^−1^) [51] and C-H bonds (2900–3000 cm^−1^) [52,53]. The presence of C-H bonds is due to the presence of organic compounds due to incomplete combustion of the pomace, as indicated by LOI data (Table 1). Additionally, the band centered between 1630 and 1648 cm^−1^ corresponds to the H-O-H bending vibrations of the bound water molecules and C=C bonds present in organic compounds [54]. These bands can be attributed to the presence of water molecules adsorbed on the surface or confined within the cavities of the geopolymer gel [51] and organic matter. The intensity of these bands decreases when KOH is used as activator and when lower proportions of activator (30 wt.% OPFA) are used. This suggests a reduction in the degree of adsorption of water molecules and less gel formation in KOH-activated binders and in those with lower OPFA content or a lower amount of organic matter.

In the cements, bands centered at 1409–1412 cm^−1^ for OPFA-activated cements and centered at 1420–1426 cm^−1^ for KOH-activated cements, as well as a band centered at 860–875 cm^−1^, characteristic of the asymmetric tension vibration of the C-O bonds and the deformation vibration of the carbonate groups, respectively, are observed [55]. This is due to the presence of carbonates in the OPFA activator and the reaction of excess calcium and potassium with atmospheric CO_2_. In OPFA-activated cements, a characteristic splitting of the bands centered at 1410 cm^−1^ is observed, with the appearance of a shoulder at 1457 cm^−1^. This phenomenon suggests the presence of ionic complexes in which K⁺ and Ca^2^⁺ cations coexist. The interaction of a divalent cation, such as Ca^2^⁺, with the carbonate ion (CO_3_^2−^) induces a band of higher energy compared to that corresponding to the K^+^ [56]. This unfolding could be associated with a local association between Ca^2^⁺ and K⁺ cations with the CO_3_^2−^ ion, resulting in the simultaneous formation of compounds such as calcite and potassium carbonate, according to XRD data.

## 4. Conclusions

Olive pomace fly ash (OPFA), a residue from olive oil production, has been used as an alkaline activator (30–50 wt%) of electric arc furnace slag (EAFS)-based cements cured at 80 °C for 7 days. Potassium salts present in the OPFA residue play a key role in the alkaline activation of the EAFS precursor by contributing to the increase in hydraulicity. The results indicate that the physical and mechanical properties depend on the amount of OPFA incorporated. Optimum properties are obtained with the incorporation of 40 wt% OPFA, reaching flexural and compressive strengths of 5.7 and 20.0 MPa, respectively, after 28 days of curing. The incorporation of more than 40 wt% OPFA causes a decrease in mechanical properties, attributable to excess alkali and the presence of unreacted particles in the microstructure. While lower amounts of OPFA, 30 wt%, are insufficient to fully activate the EAFS precursor, at a lower OPFA ratio, the unreacted particles have a more limited filling effect. This reduces their ability to improve matrix cohesion and decrease overall porosity. The mechanical properties were superior to those obtained using 4 M and 8 M KOH solutions, reaching maximum flexural and compressive strengths of 4.4 and 9.5 MPa for EAFS/KOH-8M cements, due to the higher amount of unreacted EAFS particles and less gel formation.

Therefore, the use of wastes such as OPFA as an alkaline activator for EAFS-based cements promotes the valorization of agro-industrial by-products, reducing dependence on commercial activators such as KOH, whose production is energy intensive and generates significant CO_2_ emissions due to the electrochemical and thermal processes necessary to obtain them, reducing the cost and environmental impact associated with cement production.

## Figures and Tables

**Table 1:** Chemical composition of raw materials.

		Chemical Composition (wt%)											
Raw Materials	SiO_2_	Al_2_O_3_	Fe_2_O_3_	CaO	MgO	K_2_O	SO_3_	MnO	Na_2_O	TiO_2_	P_2_O_5_	Cl	LOI *
**EAFS**	17.29	10.71	24.16	30.89	2.63	0.03	0.28	5.68	0.16	0.79	0.41	-	5.39
**OPFA**	1.86	0.38	0.67	5.33	0.81	52.1	6.81	0.02	0.19	0.05	1.62	4.14	24.90

* LOI: Loss of ignition.

**Table 2:** Composition of cements and SiO_2_/Al_2_O_3_, K_2_O/SiO_2_ molar ratio.

Cement	EAFS (g)	OPFA (g)	KOH (g)	H_2_O (g)	SiO_2_/Al_2_O_3_ (mol/mol)	K_2_O/SiO_2_ (mol/mol)
**EAFS/30OPFA**	280	120	-	104	2.83	0.79
**EAFS/40OPFA**	240	160	-	104	2.88	1.20
**EAFS/50OPFA**	200	200	-	104	2.94	1.73
**EAFS/KOH-4M**	372	-	28	104	2.74	0.23
**EAFS/KOH-8M**	344	-	56	104	2.74	0.25

**Table 3:** Bulk density, water absorption, total porosity and degree of reaction of the binders as a function of curing time.

Cement	Curing Time (days)	Bulk Density (kg/m^3^)	Water Absorption (%)	Total Porosity (%)	Degree of Reaction (%)
**EAFS/30OPFA**	7 d	1807 ± 18	15.13 ± 0.42	35.69 ± 0.51	89.5
	28 d	1830 ± 15	12.99 ± 0.57	34.25 ± 0.97	89.7
**EAFS/40OPFA**	7 d	1840 ± 17	8.97 ± 1.03	26.85 ± 1.67	91.0
	28 d	1879 ± 30	8.18 ± 0.45	24.54 ± 0.68	91.6
**EAFS/50OPFA**	7 d	1812± 29	11.16 ± 0.37	29.85 ± 0.50	90.7
	28 d	1840 ± 20	9.44 ± 0.73	27.62 ± 1.10	90.9
**EAFS/KOH-4M**	7 d	1906 ± 14	15.37 ± 0.20	27.33 ± 0.41	89.1
	28 d	1940 ± 33	14.34 ± 0.98	25.04 ± 1.03	89.3
**EAFS/KOH-8M**	7 d	2023 ± 15	13.68 ± 0.42	26.14 ± 0.65	89.5
	28 d	2039 ± 29	11.51 ± 0.36	23.45 ± 0.64	89.9

## Data Availability

The original contributions presented in this study are included in the article material. Further inquiries can be directed to the corresponding author.

## Abbreviations

The following abbreviations are used in this manuscript:

EAFS	Electric arc furnace slag
OPFA	Olive pomace fly ash
OBA	Olive pit ash
BFS	Black furnace slag
OBFA	Olive biomass fly ash
OBBA	Olive biomass bottom ash
CC	Calcined clays
GGBS	Ground granulated blast-furnace
XRD	X-ray diffraction
SEM	Scanning electron microscopy
EDS	Energy dispersive X-ray spectroscopy
XRF	X-ray fluorescence
FTIR	Fourier transform infrared spectroscopy
ATR	Attenuated total reflection
ABA	Almond shell biomass
